# Iodine Deficiency and Excess in Brazilian Pregnant Women: A Multicenter Cross-Sectional Study (EMDI-Brazil)

**DOI:** 10.3390/nu17172753

**Published:** 2025-08-26

**Authors:** Aline Carare Candido, Francilene Maria Azevedo, Sarah Aparecida Vieira Ribeiro, Anderson Marliere Navarro, Mariana de Souza Macedo, Edimar Aparecida Filomeno Fontes, Sandra Patricia Crispim, Carolina Abreu de Carvalho, Nathalia Pizato, Danielle Góes da Silva, Franciane Rocha de Faria, Jorge Gustavo Velásquez Meléndez, Barbosa Míriam Carmo Rodrigues, Naiara Sperandio, Renata Junqueira Pereira, Silvia Eloiza Priore, Sylvia do Carmo Castro Franceschini

**Affiliations:** 1 Postgraduate Program in Nutrition Science, Department of Nutrition and Health, Federal University of Viçosa (UFV), Viçosa 36570-900, Minas Gerais, Brazilsylvia@ufv.br (S.d.C.C.F.);; 2 Department of Health Sciences, Faculty of Medicine, Ribeirão Preto Medical School (FMRP), University of São Paulo (USP), Ribeirão Preto 05508-090, São Paulo, Brazil; 3 Department of Nutrition, Federal University of the Jequitinhonha and Mucuri Valleys (UFVJM), Diamantina 39100-000, Minas Gerais, Brazil; 4 Department of Food Technology, Federal University of Viçosa (UFV), Viçosa 36570-900, Minas Gerais, Brazil; 5 Postgraduate Program in Food and Nutrition, Department of Nutrition, Federal University of Paraná (UFPR), Curitiba 80210-170, Paraná, Brazil; 6 Department of Public Health, Federal University of Maranhão (UFMA), São Luís 65020-070, Maranhão, Brazil; 7 Postgraduate Program in Human Nutrition, Department of Nutrition, University of Brasília, Brasília 70970-000, Brazil; 8 Postgraduate Program in Nutrition Sciences, Department of Nutrition, Federal University of Sergipe (UFS), Aracaju 49060-025, Sergipe, Brazil; 9 Department of Medicine, Federal University of Rondonópolis (UFR), Rondonópolis 78736-900, Mato Grosso, Brazil; 10 Graduate Program in Nursing, School of Nursing, Federal University of Minas Gerais, Belo Horizonte 30130-100, Minas Gerais, Brazil; 11 Postgraduate Program in Nutrition and Health, Department of Nutrition and Health, Federal University of Espírito Santo (UFES), Vitória 29075-910, Espírito Santo, Brazil; 12 Institute of Food and Nutrition, Federal University of Rio de Janeiro (UFRJ), UFRJ Multidisciplinary Center, Macaé 21941-901, Rio de Janeiro, Brazil; 13 Department of Nutrition, Federal University of Tocantins, Palmas 77001-090, Tocantins, Brazil; renatajunqueira@uft.edu.br

**Keywords:** iodine, nutritional status, pregnancy, prenatal care, Brazil

## Abstract

**Background/Objectives:** Iodine is an important nutrient for the human body, used in the production of thyroid hormones. During pregnancy, a deficiency can cause miscarriage and hypothyroidism, while an excess can cause thyroid dysfunction. Therefore, the objective of this study was to evaluate the factors associated with the iodine nutritional status of pregnant Brazilian women. **Methods:** This was a cross-sectional, multicenter study conducted with pregnant women over 18 years of age, users of the Unified Health System (SUS). A semi-structured questionnaire was used to obtain sociodemographic information. Iodine status was assessed by urinary iodine concentration (UIC). The iodine content of salt and homemade and industrial seasonings was determined by the titrimetric method. Dietary intake was estimated through a 24-hour dietary recall. The chi-square test and hierarchical multinomial logistic regression were used for statistical analysis. The significance level was set at *p* ≤ 0.05. **Results:** Among Brazilian pregnant women, the median UIC was 186.7 µg/L (P25: 118.05 µg/L-P75: 280.93 µg/L). Regarding iodine nutritional status, the prevalence of deficiency was 36.7% (n = 694), above the requirement was 28.7% (n = 543), and excess iodine intake was 3.6% (n = 68). We observed that non-white pregnant women were more likely (OR = 1.83; 95% CI: 1.27–2.64) to have iodine deficiency, and those who did not work were less likely (OR = 0.71; 95% CI: 0.52–0.98). Pregnant women in the last trimester of pregnancy were less likely to have iodine intake above the requirements (OR = 0.52; 95% CI: 0.31–0.88). **Conclusions:** A substantial proportion of pregnant women had iodine deficiency or intake above the required level. Iodine deficiency is more chance among non-white pregnant women and less chance among those not employed during pregnancy. On the other hand, pregnant women who were in their third trimester of pregnancy were less likely to have iodine intake above the required level.

## 1. Introduction

Iodine is a nutrient used in producing the thyroid hormones triiodothyronine (T3) and thyroxine (T4), which are essential for basal metabolism, bone growth, and central nervous system development during pregnancy and, especially, in a child’s first years [1]. Low iodine intake can cause a deficiency that reduces the production and activity of these hormones, potentially leading to spontaneous abortions and hypothyroidism during pregnancy [2]. In children, it can result in low birth weight as well as physical, neurological, and intellectual issues [3,4].

During the first trimester, which runs from conception to the 13th week of gestation, the fetus relies on maternal iodine reserves to produce thyroid hormones [5]. From the 14th week, known as the second trimester, which extends to the 27th week, until the end of gestation, the fetal thyroid already has reserves to synthesize the baby’s thyroid hormones [6].

The daily iodine intake requirement during pregnancy is 250 µg; however, meeting this amount can be challenging since iodine is found in small quantities in food. Additionally, the physiological and biological changes that occur during pregnancy, such as increased glomerular filtration rate and the transfer of thyroid hormones to the fetus—leading to iodine loss—make pregnant women more vulnerable to iodine deficiency, which can cause Iodine Deficiency Disorders (IDDs). Despite this, iodine supplementation for pregnant women is not routine in Brazil [7,8,9].

In this context, universal salt iodization has been adopted worldwide, being mandatory in 123 countries, such as Switzerland, the United States, Uganda, Kenya, the Democratic Republic of the Congo, and Ethiopia, and voluntary in 21 countries, such as Portugal [10]. In Brazil, salt iodization for human consumption has been mandatory since December 1974, and the current law requires that 1 kg of salt contain between 15 and 45 mg of iodine [11]. This public policy is cost-effective and efficient, as it has helped improve iodine nutrition status in various parts of the world [12,13].

However, even with progress, iodine deficiency remains a public health problem worldwide, affecting 60% of pregnant women [14]. Excessive intake, in turn, which can result from a diet or groundwater naturally rich in iodine, can cause goiter, thyroid autoimmunity, and dysfunctions such as hyperthyroidism and hypothyroidism [5,15].

Iodine nutritional status during pregnancy can be influenced by sociodemographic conditions, such as household crowding, paid work, schooling, and the percentage of income spent on food, which affect food access and availability, which are directly related to adequate consumption of this micronutrient [16].

Therefore, a multicenter study covering the North, Northeast, South, Southeast, and Central-West regions is the first conducted in Brazil that will contribute to the diagnosis of iodine nutritional status during pregnancy, which will be important for directing public health interventions. The objective of this study was to evaluate the factors associated with the iodine nutritional status of pregnant Brazilian women.

## 2. Materials and Methods

### 2.1. Study Design and Population

This is a cross-sectional study of Brazilian pregnant women. This research is part of the Multicenter Study of Iodine Deficiency (EMDI-Brazil), which aimed to assess the nutritional profile of iodine, sodium, and potassium in the maternal and infant population.

The research centers were selected based on the institutional infrastructure, all of which were linked to higher education institutions and had researchers with recognized expertise in the field.

EMDI-Brazil was carried out in research centers in 11 municipalities in nine states and the Federal District. The municipalities investigated were Palmas (TO), Aracajú (SE), São Luís (MA), Macaé (RJ), Belo Horizonte (MG), Viçosa (MG), Vitória (ES), Ribeirão Preto (SP), Rondonópolis (MT), Brasília (DF), and Pinhais (PR).

### 2.2. Ethical Aspects

This study was approved by the Human Research Ethics Committee of the Federal University of Viçosa (UFV) under no. Register 2.496.986 and by the Human Research Ethics Committees of all the institutions involved in the study. Data collection was conducted only after participants had authorized and signed the Informed Consent Form (ICF). After the interview, all pregnant women received guidance on the importance of iodine for their health and that of their children.

### 2.3. Sample Calculation

For the sample calculation, a simple random sample was defined, with an estimated prevalence of 50%, a margin of error of 5%, and a confidence interval of 95%, resulting in 177 individuals. After adding 10% for possible losses, the sample size was adjusted to 195 pregnant women. This sample size was the same for all study centers, resulting in a final sample of 2145 pregnant women.

In some of the centers where the study was conducted, more than 195 participants were included, because concurrently with this study, other studies were being carried out with different objectives that would use the same sample. On the other hand, some centers did not reach the defined sample size due to data collection interruptions caused by the COVID-19 pandemic.

Thus, data were collected from 273 (11.5%) pregnant women in Aracajú (SE), 202 (8.5%) in Brasília (DF), 162 (6.8%) in Belo Horizonte (MG), 220 (9.3%) in Macaé (RJ), 93 (3. 9%) in Palmas (TO), 282 (11.9%) in Pinhais (PR), 278 (11.7%) in Ribeirão Preto (SP), 235 (9.9%) in Rondonópolis (MT), 299 (12.6%) in São Luís (MA), 272 (11.4%) in Viçosa (MG) and 60 (2.5%) in Vitória (ES).

The sample size was calculated to meet other research objectives. Thus, to assess iodine nutritional status, the sample power was calculated, considering sample losses and as not all pregnant women provided urine samples for evaluation. To calculate the sample power for cross-sectional studies, OpenEpi version 3.01 was used. The exposure variable considered was race (white/non-white), since being non-white was identified as a risk factor for iodine deficiency (outcome). After the calculation, considering a 95% confidence interval, the power obtained was 93.6%.

### 2.4. Inclusion and Non-Inclusion Criteria

Pregnant women using the public health network were considered eligible. Pregnant women under the age of 18, with a history of thyroid disease and/or surgery, reported diagnosis of hypothyroidism or hyperthyroidism, previous hypertension, or hypertensive pregnancy syndrome were not included.

### 2.5. Data Collection

Data were gathered in a single interview with pregnant women who were in their first, second, or third trimester. At each location studied, there was a team responsible for the collection that took place between October 2018 and January 2021. The first contact with the pregnant woman was made at each of the municipalities’ Basic Health Units (UBSs), where any doubts about the project were clarified and signatures were collected. A semi-structured questionnaire was then administered to collect socioeconomic, demographic, environmental, and health information from the pregnant women. This questionnaire was constructed and applied in person using the online data collection and management platform: Research Electronic Data Capture (REDCap^®^) version 7.0.

The questionnaire was subdivided into five parts, the first consisting of questions related to age, trimester of pregnancy, which was self-reported and then checked by the date of the last menstrual period reported, and history of thyroid disease, classifying the pregnant woman as eligible or ineligible. In the event of ineligibility, the interview was terminated, and the pregnant woman was given nutritional advice and a folder highlighting the importance of iodine for her health and that of her child.

### 2.6. Urine Collection and Analysis

In order to characterize the iodine nutritional status of pregnant women, the urinary iodine concentration (UIC) was determined. This is recommended as the most sensitive biochemical indicator of iodine deficiency and a nutritional marker of recent dietary intake.

During the interview, the pregnant women were instructed on the procedures for collecting and packaging the urine samples. The standardized procedure involved collecting 10 mL of casual urine in a sterile, hermetically sealed, and previously identified container. The samples were separated into 5 mL aliquots and stored at −20 °C in the respective collaborating centers until they were sent to the Clinical and Toxicological Analysis Laboratory of the Faculty of Pharmaceutical Sciences of the University of São Paulo (USP) in Ribeirão Preto.

The analysis was carried out using an Elan DRC II inductively coupled plasma mass spectrometer (ICP-MS) (Perkin-Elmer, Norwalk, CT, USA) operating with high-purity argon (99.999%, White Martins, Rio de Janeiro, Brazil). ICP-MS offers robustness, precision, specificity, speed, and the ability to be coupled with multielement analyses. Due to this specificity, ICP-MS is not only a resource for quality assurance but is also particularly adaptable for long-term monitoring of the population’s iodine status.

For sample preparation and ICP-MS analysis, the method proposed by Marcus et al. (2008) was used, with some modifications, in which 500 μL of each urine sample was diluted with 9 mL of solution containing 1% (*v*/*v*) Tetramethylammonium Hydroxide (TMAH) + 0.01% Triton X-100. Calibration curves were prepared in bovine base urine under the same conditions as the samples [17]. Quality control was carried out using certified urine reference material from the National Institute of Standards and Technology (NIST), SRM 2670a—Toxic Elements in Freeze-Dried Urine.

UIC was classified according to the epidemiological criteria defined by the World Health Organization, where a urinary excretion below 150 µg/L reflects insufficient iodine intake in the pregnant women group.

A median value between 150 and 249 µg/L indicates adequate iodine intake; between 250 and 499 µg/L means above requirement; and a median above 500 µg/L indicates excessive iodine intake [7].

In the study, our sample size calculation determined that 2145 pregnant women should be evaluated, but 2376 women were interviewed, as this sample was also used for other research purposes. However, there were sample losses, as of the 2376 pregnant women interviewed, 485 did not send urine samples. The collection team attempted to contact these participants on three separate occasions to encourage them to send samples, but without success. Therefore, the universe for analysis of iodine nutritional status was reduced by 20.4% (n = 1891).

### 2.7. Collection and Analysis of Salt and Seasonings

Iodine availability in foods was assessed by analyzing the iodine content in samples of salt consumed at home and in alternative sources of iodine, including pure iodine, such as industrial or homemade seasonings. Samples of salt, industrial, or homemade seasonings were collected from a 20% (n = 378) subsample of pregnant women who provided urine samples (n = 1891) during a home visit.

Approximately 50 grams of household salt and 20 grams of industrial or homemade seasonings were collected in a hermetically sealed and previously identified plastic container. The salt samples were stored in a sealed container in a dry, well-ventilated place until they were analyzed, and the seasoning samples were frozen at −20 °C until they were sent to the Food Chemistry and Analysis Laboratory of the Food Technology Department at the Federal University of Viçosa (UFV).

However, the evaluation of consumer salt and seasonings was not conducted at the Brasília, Macaé, and Palmas centers due to collection interruptions caused by the COVID-19 pandemic.

To assess the iodine content in salt, the titrimetric method recommended by the Ministry of Health was used and analyzed according to the Adolfo Lutz Institute manual [18]. Samples with an iodine content of between 15 and 45 mg/kg of salt were considered adequate, as recommended by Resolução da Diretoria Colegiada (RDC) no. 23 of April 24, 2013, of the National Health Surveillance Agency [19]. The Moxon and Dixon method, adapted by Perring et al., was initially used to assess iodine in seasonings in triplicate, blindly and randomly [20,21].

### 2.8. Food Consumption Assessment

To estimate the dietary intake of pregnant women, during the interview at the UBS, a 24-hour recall (24HR) was applied to the entire sample, followed by the application of a second recall to a sub-sample of 18.3% (n = 412). The multiple-step method was used to conduct the interview, and the Brazilian Food Quantification Manual was used to quantify the food portions of the 24HR [22,23]. The Table of Iodine Composition in Foods (TCIA) was used to estimate iodine intake. Iodine composition data from other tables were consulted when not available in the TCIA [24].

During the interview, pregnant women were asked about their use of iodine-containing nutritional supplements. However, the amount of iodine present in the supplement was not incorporated into the quantification of dietary iodine intake, as the prevalence of iodine supplement use was very low (6.7%; n = 122) in our sample. Therefore, we chose to consider only the iodine consumed by them through diet.

The University of California, Davis (UCD)/National Cancer Institute (NCI) Simulating intake of micronutrients for Policy Learning and Engagement (SIMPLE) tool was used to quantify habitual iodine intake and the prevalence of inadequate intake, minimizing the effect of intraindividual variability on these estimates [25,26].

The adequacy of dietary iodine intake was based on the harmonized intake reference values proposed for populations, in order to allow comparison of the results between different epidemiological contexts [27]. Thus, the average requirement (H-AR < 160 µg) and upper-level intake (H-UL > 600 µg) values were used to estimate the percentage of pregnant women with insufficient and excessive intake, respectively [27].

Intake analyses were carried out for the total sample. SAS OnDemand for Academics version 3.1.0 (SAS Institute Inc., Cary, NC, USA) [28] was used to estimate habitual iodine intake.

### 2.9. Statistical Analyses

The databases were exported from the REDCap^®^ software to the Statistical Package for Social Sciences (SPSS) version 21.0. The Shapiro–Wilk test was applied to check the distribution pattern of the quantitative variables, followed by visual analysis using a histogram. Absolute and relative frequencies and measures of central tendency and dispersion were presented, using the mean with standard deviation or confidence interval (95%CI) for parametric variables and the median with interquartile range or 25th and 75th percentiles for non-parametric variables.

Pearson’s chi-square test was used to verify the association between obstetric and socioeconomic variables and UIC, with a 95% confidence interval, considering the extremes of urinary excretion, which was categorized into the 25th percentile, which comprised values ≤ 118.05 µg/L, and the 75th percentile, with values ≥ 280.93 µg/L.

**Figure 1:**
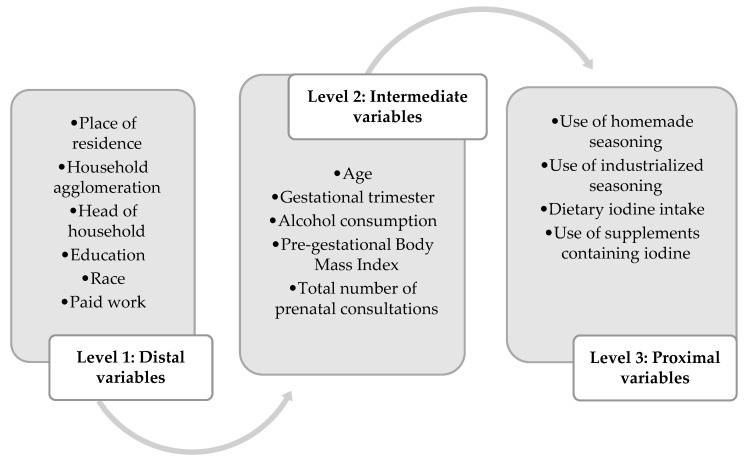
Hierarchical theoretical model used in multinomial logistic regression, since urinary iodine concentration, which is our dependent variable, is in ordinal form. Thus, the variables associated with the iodine nutritional status of Brazilian pregnant women were separated by level of contribution to iodine nutritional status, and segmented into: level 1 (proximal): sociodemographic variables, which can indirectly influence iodine nutritional status; level 2 (intermediate): health, lifestyle, and obstetric variables of the pregnant woman; level 3 (distal): variables directly related to iodine nutritional status, which include the use of spices, food consumption and iodine supplementation during pregnancy. Viçosa, 2019–2021.

The Statistical Software (STATA) version 14.0 was also used for the multinomial logistic regression analysis to assess the factors associated with iodine nutritional status, adding the variables by levels, according to the hierarchical theoretical model. At level 1, sociodemographic variables were considered, which can indirectly influence iodine nutritional status. At level 2, variables related to the pregnant woman’s health, lifestyle, and obstetric characteristics were added. Finally, level 3 included variables directly related to iodine nutritional status, as shown in Figure 1.

The food consumption variable used in the analysis included the amount of iodine ingested through water, salt, spices, and other food sources. Although the number of spices used was included in the iodine dietary intake variable, the variable indicating whether the pregnant woman used homemade or industrialized spices was also included among the proximal variables, as it may be that on the day the recall was carried out, the pregnant woman did not use spices when preparing food. In addition, information on alcohol consumption was included in level 2, as alcohol reduces iodine absorption, increases its urinary excretion, and interferes with thyroid metabolism (Figure 1).

For analysis, urinary iodine was classified into three categories: deficiency (urinary excretion below 150 µg/L), adequate (urinary excretion between 150 and 249 µg/L), and above requirement (urinary excretion between 250 and 499 µg/L) [7]. Variables with a *p*-value ≤ 0.20 at their level were included at the next level in order to identify which of the variables in the theoretical model were potential risk factors.

The effect of each variable on the outcome was assessed separately at its level, and variables with a *p*-value ≤ 0.05 were considered associated in the final model. Odds ratio (OR) values are presented with 95% confidence intervals (95% CIs).

## 3. Results

Brazilian pregnant women had a median UIC of 186.7 µg/L (P25: 118.05 µg/L-P75: 280.93 µg/L), classified as adequate according to WHO epidemiological criteria. Nationally, the prevalence of iodine deficiency was 36.7% (n = 694), that of adequacy was 31% (n = 586), the level above the requirement was 28.7% (n = 543), and that of excess was 3.6% (n = 68).

Table 1 shows a higher proportion of UIC at the 25th percentile (≤118.05 µg/L) among pregnant women who were not white (77.9%), who lived in rural areas (6.8%), and who had seven or more consultations (23.6%). There was also a higher proportion of UIC at the 75th percentile (≥280.93 µg/L) among pregnant women who worked during pregnancy (62.2%), who lived with three or more people in the household (73.6%), and among primiparous women (68.7%).

The sociodemographic and health variables of pregnant women were assessed according to the classification of urinary iodine as deficient or above the required level, and we observed that, at level 1, non-white pregnant women (black, brown, indigenous, and Asian) had a higher chance of iodine deficiency when compared to white pregnant women (OR = 1.50; 95% CI: 1.16–1.94). Pregnant women who did not work during pregnancy were more likely to have an iodine nutritional status above the required level (OR = 1.30; 95% CI: 1.01–1.66) when compared to pregnant women who worked (Table 2).

At level 2, where health and obstetric variables were taken into account, pregnant women of non-white race (black, brown, indigenous and oriental) had a higher chance of iodine deficiency (OR = 1.77; 95%CI: 1.28–2.44) and pregnant women who were in the third trimester of pregnancy had a lower chance of iodine nutritional status above the requirement (OR = 0.64; 95%CI: 0.41–0.99) (Table 3).

In the final model, non-white pregnant women were more likely to have iodine deficiency (OR = 1.83; 95% CI: 1.27–2.64) compared to white pregnant women, and those who did not work (OR = 0.71; 95% CI: 0.52–0.98) were less likely than those who worked. Pregnant women in the last trimester of pregnancy were less likely to have iodine intake above their requirements (OR = 0.52; 95% CI: 0.31–0.88) (Table 4).

## 4. Discussion

Considering the median UIC, Brazilian pregnant women presented an adequate iodine nutritional status. However, this result cannot be evaluated in isolation, as when we assess nutritional status, we observe a significant prevalence of deficiency and intake above the requirement.

In nutritional care provided to pregnant women, when iodine nutritional status is discussed, there is a lack of knowledge about the importance of this micronutrient. This reinforces the need for health education initiatives to raise awareness among mothers about the importance of iodine for their health and that of their children, about the correct storage of salt, which is the main dietary source of this nutrient, and to reinforce the importance of an adequate diet based on natural foods, such as fruits and vegetables, which support the intake of important micronutrients during this phase, and avoiding ultra-processed foods that are harmful to health.

Currently, the Brazilian population is classified as having adequate iodine intake, based on the report of the 2016 National Survey to Assess the Impact of Salt Iodization (PNAISAL), which assessed 19,600 schoolchildren aged 6 to 14 years [13,29,30]. However, it is important to note that this assessment may not represent the maternal and child group, due to the differences in physiology, intake needs, and food consumption between these groups.

In our study, non-white women were more likely to have iodine deficiency. The influence of this variable on maternal iodine nutritional status may be mediated by racial inequality, which may indirectly determine nutritional care, including access to dietary sources of iodine, as well as inadequate practices of consuming iodized salt [31,32,33,34]. The white race/color is privileged because it has less exposure to discriminatory events and greater access to education, health, and employment, which distances contact from the agents that cause malnutrition, nutritional deficiencies, and hidden hunger, which are determining factors in the health-disease process [35].

We also found that pregnant women who worked during pregnancy were less likely to be iodine-deficient. The median household income among non-working pregnant women was R$1200.00 compared to R$2300.00 for pregnant women who worked. Individuals with lower incomes consume more ultra-processed foods due to their low price and ease of purchase [36,37]. These products have a high caloric density, are rich in sugars, fats, and sodium, and are formulated with iodized salt, which can act as a protective factor against deficiency [38].

Furthermore, pregnant women living in rural areas had a UIC in the 25th percentile. Rural residence hinders the use of iodized salt for food preparation due to unfamiliarity with the product, substitution by natural seasonings, and difficulties in accessing urban areas to purchase salt, caused by long distances and poor road quality [16,39]. These factors negatively affect the adequacy of iodine nutritional status in this population.

In a study carried out in Novo Cruzeiro, a semiarid region of Minas Gerais, Brazil, which evaluated the nutritional status of iodine in schoolchildren, the authors observed a higher prevalence of deficiency in rural areas, being three times more frequent in the rural population compared to the urban population (27.1 and 9.4%, respectively) and there was a 2.12 times greater chance of iodine deficiency (1.58 < OR = 2.12 < 2.84) [40].

However, due to the differences between urban and rural populations, which include sanitation conditions and access to health care services, the location of the household may be an important confounding factor in accurately understanding the distribution of iodine deficiency and its determining factors.

There was also an association between parity and UIC at the 25th percentile, with a higher proportion among multiparous women. Studies have shown that multiparity associated with an inadequate diet can cause vitamin and mineral deficiencies, because the body’s micronutrient reserves, such as iodine, even if small, are replenished in the non-pregnant period, so women with an interpartal interval of less than two years are more likely to develop iodine deficiency in pregnancy later on [41,42].

The Brazilian population is currently using salt on a large scale. According to data from the National Health Survey (PNS/IBGE), the average per capita salt consumption of adults is 9.3 g/day, almost double the WHO recommendation of 5 g/day, and only 14.2% recognize their salt consumption as Excessive [43,44]. However, it is important to note that salt added during cooking is declining, while salt intake from processed foods is increasing, especially in metropolitan areas [45].

Excess iodine in the body is a concern because it inhibits iodine uptake, thyroglobulin iodination, and the release of thyroid hormones, resulting in the temporary inhibition of thyroid hormone synthesis [46,47]. In our study, pregnant women in the last trimester were less likely to have iodine intake above their needs. This may occur because during this period, there is uterine expansion caused by the baby’s growth and weight gain, leading to bladder compression and increased urinary frequency, which, combined with increased glomerular filtration caused by the expansion of blood volume, leads to loss of iodine in the urine [48].

In addition, there was an association between UIC at the 75th percentile and household crowding, with a higher proportion among pregnant women who lived with three or more people. Greater household crowding is usually associated with lower income, which implies greater consumption of ultra-processed foods, which contain iodine [49,50].

There was also an association between the total number of prenatal consultations and UIC at the 75th percentile. Prenatal care allows for maternal dietary counseling, promoting the prevention of obstetric problems that can affect the health of the mother and child; however, this work has a long-term effect, with a greater number of appointments [51].

Among the strengths of the study, we highlight the novelty of analyzing the iodine concentration of different sources, such as water, seasonings, food, and salt, to measure the iodine nutritional status of Brazilian pregnant women, with national representation.

The limitations of this study were the random application of the 24 h dietary recall, which may have reduced the capture of sporadic consumption of important dietary sources of iodine, such as fish. Furthermore, the iodine concentration in foods was biased by the use of nutritional composition information from composition tables from other countries. Therefore, as a suggestion for future studies, we recommend that a Brazilian iodine-source Food Composition Table be drawn up and the analysis of the iodine nutritional status of other physiological groups.

## 5. Conclusions

Pregnant Brazilian women presented adequate iodine nutritional status, but with a significant prevalence of deficiency and intake above the required level, reflecting socioeconomic and health inequalities among Brazilian women.

We observed a higher risk of iodine deficiency among non-white pregnant women and a lower risk among pregnant women who did not work during pregnancy. Women in their third trimester of pregnancy were also less likely to have iodine intakes above the required level.

In this context, it is necessary to develop manuals for primary health care on the importance of iodine during pregnancy and in the first years of a child’s life. Finally, our results reinforce the importance of including pregnant women in the National Program for Periodic Monitoring of Iodine Nutritional Status in Brazil and the need to reformulate the current iodization range.

## Figures and Tables

**Table 1:** Association between urinary iodine concentration and obstetric and socioeconomic variables of Brazilian pregnant women. EMDI-Brazil, 2019–2021.

	Urinary Iodine Concentration			
Variables	25th Percentile N (%)	75th Percentile N (%)	Total N (%)	*p*-Value
Race				0.002 **
White	104 (22.1%)	148 (31.2%)	600 (26.8%)	
Non-white *	367 (77.9%)	326 (68.8%)	1636 (73.2%)	
Paid work				0.010 **
Yes	218 (46.3%)	179 (37.8%)	961 (43.0%)	
No	253 (53.7%)	294 (62.2%)	1274 (57.0%)	
Total number of prenatal consultations				0.002 **
7 appointments or more	104 (23.6%)	68 (15.1%)	389 (18.3%)	
Up to 6 visits	336 (76.4%)	382 (84.9%)	1731 (81.7%)	
Place of residence				0.043 **
Urban	440 (93.2%)	456 (96.2%)	2117 (94.6%)	
Rural	32 (6.8%)	18 (3.8%)	122 (5.4%)	
Household crowding				0.048 **
Up to 2 people in the household	150 (32.3%)	124 (26.4%)	649 (29.3%)	
3 or more people in the household	315 (67.7%)	346 (73.6%)	1569 (70.7%)	
Parity				0.027 **
Nulliparous	143 (59.1%)	184 (68.7%)	747 (57.5%)	
Multiparous	99 (40.9%)	84 (31.3%)	552 (42.5%)	

* Black, brown, indigenous, and oriental. Pearson’s chi-square test. EMDI: Multicenter Iodine Deficiency Study. ** Significant result: *p* < 0.05.

**Table 2:** Sociodemographic characteristics of Brazilian pregnant women (level 1) according to the classification of urinary iodine in deficiency and above requirement, compared to the adequate category. EMDI-Brazil, 2019–2021.

	Deficiency			Above Requirement		
Variables	n (%)	OR (95%CI)	*p*-Value	n (%)	OR (95%CI)	*p*-Value
Place of residence						
Urban	640 (37.4%)	Ref.	Ref.	519 (30.4%)	Ref.	Ref.
Rural	43 (42.2%)	1.09 (0.68–1.76)	0.695	24 (23.5%)	0.66 (0.38–1.15)	0.149
Household agglomeration						
Up to 2 people in the household	213 (40.5%)	Ref.	Ref.	143 (27.2%)	Ref.	Ref.
3 or more people in the household	460 (36.2%)	0.89 (0.70–1.15)	0.417	396 (31.2%)	1.14 (0.87–1.49)	0.310
Head of household						
Pregnant woman	182 (37.6%)	Ref.	Ref.	147 (30.4%)	Ref.	Ref.
Other	502 (37.8%)	1.01 (0.78–1.32)	0.888	395 (29.8%)	1.14 (0.87–1.49)	0.310
Education						
Higher/Undergraduate	117 (40.8%)	Ref.	Ref.	83 (28.9%)	Ref.	Ref.
Elementary School	141 (35.6%)	0.92 (0.62–1.35)	0.676	138 (34.8%)	1.11 (0.74–1.67)	0.595
High School	422 (37.7%)	0.83 (0.60–1.15)	0.277	321 (28.7%)	0.83 (0.60–1.19)	0.345
Race						
White	153 (31.4%)	Ref.	Ref.	160 (32.8%)	Ref.	Ref.
Non-white *	530 (40.1%)	1.50 (1.16–1.94)	0.002 **	383 (29.0%)	1.02 (0.79–1.33)	0.843
Paid work						
Yes	314 (40.5%)	Ref.	Ref.	205 (26.5%)	Ref.	Ref.
No	369 (35.7%)	0.88 (0.69–1.11)	0.293	337 (32.6%)	1.30 (1.01–1.66)	0.038 **

Multinomial logistic regression. Quantification of the total absolute and relative frequency in the line. * Black, brown, indigenous, and oriental. ** Significant result: *p* < 0.05. OR = Odds ratio. 95%CI = 95% confidence interval. Ref = reference category. EMDI: Multicenter Iodine Deficiency Study.

**Table 3:** Sociodemographic, obstetric, and health characteristics of Brazilian pregnant women (level 2) according to the classification of urinary iodine in deficiency and above requirement, compared to the adequate category. EMDI-Brazil, 2019–2021.

			Deficiency			Above Requirement		
Variables	n Median	% p25-p75	OR (95%CI)	*p*-Value	n Median	% p25-p75	OR (95%CI)	*p*-Value
Place of residence								
Urban	640	37.4%	Ref.	Ref.	519	30.4%	Ref.	Ref.
Rural	43	42.2%	0.90 (0.52–1.55)	0.717	24	3.5%	0.80 (0.44–1.44)	0.466
Race								
White	153	31.4%	Ref.	Ref.	160	32.8%	Ref.	Ref.
Non-white *	530	40.1%	1.77 (1.28–2.44)	0.000 **	383	29.0%	1.09 (0.79–1.49)	0.583
Paid work								
Yes	314	40.5%	Ref.	Ref.	205	26.5%	Ref.	Ref.
No	369	35.7%	0.83 (0.62–1.11)	0.217	337	32.6%	1.28 (0.95–1.72)	0.099
Age (years)	25.0	22–31	1.01 (0.98–1.03)	0.342	25.0	22–31	1.00 (0.97–1.03)	0.711
Gestational trimester								
First	131	35.8%	Ref.	Ref.	119	32.5%	Ref.	Ref.
Second	254	34.2%	0.96 (0.64–1.44)	0.865	241	32.4%	0.93 (0.63–1.37)	0.743
Third	303	42.8%	1.19 (0.77–1.83)	0.420	183	25.8%	0.64 (0.41–0.99)	0.045 **
Pre-gestational Body Mass Index	24.21	21.79–28.08	0.97 (0.95–1.00)	0.149	24.76	21.67–28.73	1.01 (0.98–1.03)	0.433
Total number of prenatal consultations								
7 appointments or more	149	47.3%	Ref.	Ref.	76	24.1%	Ref.	Ref.
Up to 6 appointments	493	35.5%	0.71 (0.48–1.05)	0.091	434	31.3%	1.07 (0.69–1.65)	0.742
Alcohol consumption								
No	656	37.9%	Ref.	Ref.	516	29.8%	Ref.	Ref.
Yes	29	35.8%	1.14 (0.55–2.35)	0.712	26	32.1%	1.18 (0.58–2.40)	0.642

Multinomial logistic regression. Quantification of the total absolute and relative frequency in the line. * Black, brown, indigenous, and oriental. ** Significant result: *p* < 0.05. Ref = reference category. p25-p75 = 25th and 75th percentile. OR = Odds ratio. 95%CI = 95% confidence interval. EMDI: Multicenter Iodine Deficiency Study.

**Table 4:** Sociodemographic, obstetric, health, and food consumption characteristics of Brazilian pregnant women (level 3) according to the classification of urinary iodine in deficiency and above requirement, compared to the adequate category. EMDI–Brazil, 2019–2021.

			Deficiency			Above Requirement		
Variables	n Median	% p25-p75	OR (95%CI)	*p*-Value	n Median	% p25-p75	OR (95%CI)	*p*-Value
Race								
White	153	31.4%	Ref.	Ref.	160	32.8%	Ref.	Ref.
Non-white *	530	40.1%	1.83 (1.27–2.64)	0.001 **	383	29.0%	1.13 (0.79–1.61)	0.490
Paid work								
Yes	314	40.5%	Ref.	Ref.	205	26.5%	Ref.	Ref.
No	369	35.7%	0.71 (0.52–0.98)	0.043 **	337	32.6%	1.24 (0.88–1.73)	0.204
Gestational trimester								
First	131	35.8%	Ref.	Ref.	119	32.5%	Ref.	Ref.
Second	254	34.2%	0.79 (0.48–1.30)	0.366	241	32.4%	0.76 (0.47–1.22)	0.262
Third	303	42.8%	0.92 (0.55–1.56)	0.781	183	25.8%	0.52 (0.31–0.88)	0.016 **
Pre-gestational Body Mass Index	24.21	21.79–28.08	0.98 (0.95–1.01)	0.247	24.76	21.67–28.73	1.02 (0.99–1.05)	0.174
Total number of prenatal consultations								
7 appointments or more	149	47.3%	Ref.	Ref.	76	24.1%	Ref.	Ref.
Up to 6 appointments	493	35.5%	0.65 (0.42–1.00)	0.051	434	31.3%	1.08 (0.66–1.76)	0.757
Use of homemade seasoning								
No	456	37.2%	Ref.	Ref.	377	30.7%	Ref.	Ref.
Yes	227	39.1%	1.06 (0.76–1.48)	0.710	164	28.2%	0.96 (0.68–1.37)	0.852
Use of industrialized seasoning								
No	286	40.5%	Ref.	Ref.	206	29.1%	Ref.	Ref.
Yes	401	36.3%	0.84 (0.61–1.17)	0.325	334	30.2%	0.72 (0.52–1.01)	0.063
Dietary iodine intake (µg)	156.55	111.47–202.49	1.00 (0.99–1.00)	0.466	147.06	106.55–198.71	0.99 (0.99–1.00)	0.984
Use of supplements containing iodine								
Yes	36	37.9%	Ref.	Ref.	31	32.6%	Ref.	Ref.
No	530	38.5%	1.13 (0.60–2.11)	0.688	401	29.1%	1.09 (0.56–2.13)	0.595

Multinomial logistic regression. Quantification of the total absolute and relative frequency in the line. * Black, brown, indigenous, and oriental. ** Significant result: *p* less than 0.05. Ref = reference category. p25-p75 = 25th and 75th percentile. OR = odds ratio. 95%CI = 95% confidence interval. EMDI: Multicenter Iodine Deficiency Study.

## Data Availability

The data presented in this study will be made available upon request to the corresponding author due to privacy concerns, as it belongs to the Department of Nutrition and the Graduate Program in Nutrition Science at the Federal University of Viçosa, located in the state of Minas Gerais, Brazil.
